# Utility Of POC Xpert HIV-1 Tests For Detection-Quantification Of Complex HIV Recombinants Using Dried Blood Spots From Kinshasa, D. R. Congo

**DOI:** 10.1038/s41598-019-41963-y

**Published:** 2019-04-05

**Authors:** Marina Rubio-Garrido, Adolphe Ndarabu, Gabriel Reina, David Barquín, Mirian Fernández-Alonso, Silvia Carlos, África Holguín

**Affiliations:** 1 HIV-1 Molecular Epidemiology Laboratory, Microbiology and Parasitology Department, University Hospital Ramón y Cajal-IRYCIS and CIBEREsp-RITIP, Madrid, 28034 Spain; 2 Monkole Hospital, Kinshasa, Democratic Republic of the Congo; 3 Microbiology Department, Clínica Universidad de Navarra, Navarra Institute for Health Research (IdiSNA), Institute of Tropical Health, Universidad de Navarra (ISTUN), Pamplona, 31008 Spain; 4 Department of Preventive Medicine and Public Health, Institute of Culture and Society (ICS), Institute of Tropical Health (ISTUN), Universidad de Navarra, Navarra Institute for Health Research (IdiSNA), Pamplona, 31008 Spain

## Abstract

Point-of-Care (POC) molecular assays improve HIV infant diagnosis and viral load (VL) quantification in resource-limited settings. We evaluated POC performance in Kinshasa (Democratic Republic of Congo), with high diversity of HIV-1 recombinants. In 2016, 160 dried blood samples (DBS) were collected from 85 children (60 HIV−, 18 HIV+, 7 HIV-exposed) and 75 HIV+ adults (65 treated, 10 naive) at Monkole Hospital (Kinshasa). We compared viraemia with Cepheid-POC-Xpert-HIV-1VL and the non-POC-COBAS^®^AmpliPrep/COBAS^®^TaqMan^®^HIV-1-Testv2 in all HIV+, carrying 72.4%/7.2% HIV-1 unique/complex recombinant forms (URF/CRF). HIV-1 infection was confirmed in 14 HIV+ children by Cepheid-POC-Xpert-HIV-1Qual and in 70 HIV+ adults by both Xpert-VL and Roche-VL, identifying 8 false HIV+ diagnosis performed in DRC (4 adults, 4 children). HIV-1 was detected in 95.2% and 97.6% of 84 HIV+ samples by Xpert-VL and Roche-VL, respectively. Most (92.9%) HIV+ children presented detectable viraemia by both VL assays and 74.3% or 72.8% of 70 HIV+ adults by Xpert or Roche, respectively. Both VL assays presented high correlation (R^2^ = 0.89), but showing clinical relevant ≥0.5 log VL differences in 15.4% of 78 cases with VL within quantification range by both assays. This is the first study confirming the utility of Xpert HIV-1 tests for detection-quantification of complex recombinants currently circulating in Kinshasa.

## Introduction

The access to routine molecular tools for early infant HIV-1 diagnosis (EID) and viral load (VL) quantification in children and adults is required for an early antiretroviral treatment failure identification and the prompt linkage to care. It can reduce HIV-associated mortality and morbidity in infected populations^1–3^. However, most of 37 million HIV-infected individuals live in resource-limited countries with a high number of different circulating HIV-1 variants, high rates of infection and no or limited access to routine HIV monitoring^4,5^. These settings have insufficient access to laboratory facilities, cold-chain management shortcomings, and difficulties for plasma collection and sample transportation^5,6^.

Until now, conventional molecular tests for EID and VL needed long procedures conducted in specialized and centralized laboratory settings requiring substantial infrastructure and training, needing turnaround times of several weeks or months^7,8^. This could increase the risk of loss to clinical follow-up of patients, thus having a negative impact in the HIV treatment cascade^9,10^.

To improve the linkage to care of HIV-exposed and infected subjects, some new easy to perform molecular assays for EID and VL quantification have been developed: point-of-care or POC assays. They have emerged as potential game-changers for improving EID and antiretroviral therapy (ART) monitoring programs^11^ since they are simpler, faster (less than 2 hours), automated platforms that do not require as much infrastructure as the conventional lab-based systems^8,12^. They can be performed directly in health centers and not only in reference laboratories, which favors their use at or near the point-of-care, allowing HIV confirmation, ART initiation, or treatment or adherence interventions quickly after sampling (within about 2 hours). WHO promotes POC use for HIV diagnosis and monitoring in limited-resource settings^13^, as well as the use of dried blood spots (DBS) instead of plasma as being easier to collect and ship to centralized facilities than plasma^14,15^. However, most POC HIV assays have not yet been evaluated using well-characterized DBS panels.

HIV genetic variability can affect the success of HIV-1 detection and quantification by molecular assays^16–23^. However, the performance of most POC and non-POC assays has not been extensively evaluated testing all HIV-1 subtypes and complex recombinants present in countries with high genetic diversity and high rate of HIV infections. This is the case of Kinshasa (Democratic Republic of Congo, DRC), the epicenter of HIV-1 group M epidemic^24^, were a large number of HIV-1 recombinants are expected^24–32^. Thus, this study analyzes the efficacy of two POC techniques for EID and VL (Cepheid Xpert HIV-1 Qual and Xpert HIV-1 VL) *versus* the non-POC Roche CAP/CTM Quantitative VL test v2.0 in the same DBS panel collected from children and adults in Kinshasa, where a large diversity of HIV-1 variants co-circulate.

## Material and Methods

From April to November 2016, 160 DBS were collected at Monkole Hospital (Kinshasa, DRC) from 85 children (60 HIV-non infected, 18 HIV-positive, 7 HIV-exposed) and 75 HIV-infected adults (65 treated with clinical suspicion of treatment failure, 10 naive). DBS samples were prepared by spotting 70 µl of venous blood with micropipette, collected by venipuncture in EDTA-anticoagulant tubes into each dot on a Whatman 903 Protein Saver Card (Schleicher & Schuell, Dassel, Germany). Two or three DBS cards were collected per patient. They were dried separately on a drying-rack overnight at room temperature in Monkole Hospital, sealed in a zip-lock plastic bag with desiccant bags and stored at −20 °C until transported in dry ice to the laboratories in Madrid and Pamplona, Spain, where children and adult samples, respectively, were stored at −80 °C until further use.

### HIV diagnosis and viraemia quantification

HIV diagnosis was firstly performed in DRC using rapid serological tests: Determine™ HIV-1/2 Ag/Ab (Alere), Double-Check Gold HIV 1&2 (Orgenics) and Uni-Gold HIV (Trinity Biotech) from 18-months old and by Biomerieux 4th generation immunoassay VIDAS® HIV Duo Ultra or exceptionally by molecular Abbott real-time HIV-1 Qualitative in infants under 18-months old. In Madrid, Spain, HIV serological status in the 85 children was confirmed with BioRad Geenius^TM^ HIV-1/2 confirmatory assay using one DBS dot per patient, as previously reported^33^. All HIV seropositive and undetermined pediatric DBS by Geenius were then tested by POC Cepheid Xpert Qual (Xpert Qual), which provides a binary “detected”/“not detected” result^34^. In Navarra, Spain, HIV serostatus was confirmed in all adults by two 4th generation immunoassays: Elecsys® HIV combi PT (Roche) and VIDAS® HIV Duo Quick (bioMerieux).

HIV-1 viremia was quantified using Cepheid Xpert HIV-1 VL (Xpert VL)^35^ and COBAS® AmpliPrep/COBAS® TaqMan® HIV-1 Test v2.0 (Roche VL)^36^ in all HIV+ DBS, both techniques based on real time amplification of HIV genome. All assays were performed using one dot eluted in Xpert Qualitative buffer for Xpert assays or Roche SPEX buffer for Roche-VL as lysis buffer to elute the DBS dots, according to manufacturer’s instructions. GeneXpert® Instrument automates and integrates specimen preparation, HIV-1 total nucleic acids (viral RNA and proviral DNA) extraction and amplification, and detection of the target sequence in specimens using real-time reverse transcriptase PCR (RT-PCR). The systems require the use of single-use disposable GeneXpert® cartridges that hold all the necessary RT-PCR reagents and host the RT-PCR processes.

For statistical analysis of VL data, any viraemia values reported by the system as <40 cp/ml (by Xpert VL) or <20 cp/ml (by Roche VL), lower limit of detection of each assay, were reported as 39 cp/ml or 19 cp/ml, respectively, being considered detected but not quantifiable. We identified treated subjects under therapeutic failure when they present HIV-1 viraemias of 1,000 cp/ml or higher, clinical treatment failure threshold using DBS^37^. Both HIV-1 VL assays were based on real time PCR, providing an assay-specific cycle threshold (Ct), which inversely correlates with the starting concentration of the viral genome in the infected specimen. Ct values were recorded following DBS VL quantification by both Xpert VL and Roche VL platforms using one DBS dot in each sample.

We provided the number of HIV-1 RNA copies per dot and per plasma milliliter after considering patient’s hematocrit assuming 39% hematocrit for children, 42% for women and 47% for men, according to previous studies^38,39^. This lead to plasma volumes of 42.7 µl, 40.6 µl and 37.1 µl, respectively, in 70 µl blood collected per dot. The main features of the three molecular HIV assays used in the study are described in Table 1.

**Table 1:** Characteristics of molecular assays for HIV-1 diagnosis and VL quantification.

	Qualitative assays (for HIV-1 diagnosis)	Quantitative assays (for HIV-1 viral load quantification)	
	Xpert Qual	Roche VL	Xpert VL
Company	Cepheid	Roche	Cepheid
POC molecular assay	Yes	No	Yes
Viral targets	3′end-5′UTR	Gag + LTR	3′end-5′UTR
Sample (according to technical report)	Whole blood (100 µl) DBS (1 dot)	Plasma	Plasma (1 ml)
LOD	203 cp/ml (VQA, whole blood) 278 cp/ml (WHO, whole blood) 531 cp/ml (VQA in DBS) 668 cp/ml (WHO in DBS)	20 cp/ml (plasma)	15.3 cp/ml (VCA in plasma) 18.3 cp/ml (WHO in plasma)
LOQ	—	20 cp/ml (plasma) 20 cp/dot (DBS)*	40 cp/ml (plasma) 40 cp/dot (DBS)
Approved for EID using plasma	No	No	No
Approved for EID using DBS or whole blood	Yes	No	No
Approved for VL using plasma	—	Yes	Yes
Approved for VL using DBS	—	No	No
Detected HIV-1 groups	M, N and O	M and O	M, N and O

VL, viral load; EID, early infant HIV-1 diagnosis; POC, point of care; LOD/Q, limit of detection/quantification; DBS, Dried Blood Spots; HIV-1-RNA cp/ml, cp/ml plasma; LTR, long terminal repeats; UTR, untranslated region within viral LTR; Roche VL, COBAS® AmpliPrep/COBAS® TaqMan® HIV-1 Test v2.0; Xpert Qual, Cepheid Xpert HIV-1 Qual; Xpert VL, Cepheid Xpert HIV-1 VL; VQA: HIV-1 subtype B from viral quality assurance laboratory; WHO: HIV-1 subtype B from WHO 3^rd^ International Standard NIBSC code 10/152 http://www.nibsc.org/documents/ifu/10-152.pdf. Data according to technical reports. LOD Xpert HIV-1 VL and Xpert HIV-1 Qual available^61,76^. *Data reported by this study.

### HIV-1 variant characterization

For HIV-1 variant characterization, RNA was extracted from 2 DBS dots using the NucliSENS easyMAG automated platform (BioMerieux) or manual High-Pure Viral Nucleic Acid (Roche) kit. Viral RNA was amplified in the HIV-1 *pol* coding region by RT-PCR and nested-PCR using primers designed by WHO^40^ as previously described^41^ and/or ANRS^42^. Viral sequences included the complete HIV-1 protease (PR, codons 1–99), and partial retrotranscriptase (RT, codons 1–335/440) and integrase (IN, codons1–285). PCR amplicons were purified using the Illustra™ ExoProStar 1-Step™ (GE Healthcare Life Sciences, Little Chalfont, UK) and sequenced by Macrogen Inc. (Gasan-dong, Geumchun-gu, Seoul, Korea). HIV-1 variant was characterized by phylogenetic analysis (phy) using MEGA6 with Tamura 3-parameters as the evolutionary model with 1,000 bootstrap resampling. The bootstrap cut-off was set at 70. The tree topology was obtained using Neighbor Joining method. At least two representative HIV-1 sequences of each HIV-1 non-M group (O, P, N), and from each group M variant (9 subtypes, 6 sub-subtypes and 83 CRF available at the moment of the analysis among the 98 described^43^ were taken as references. Sequences not identified as any known non-M group, group M subtype or CRF by phy were considered HIV-1 group M unique recombinant forms (URF) in *pol* (URF*pol*).

### Accession numbers

PR, RT and/or IN HIV-1 sequences were submitted to GenBank (www.ncbi.nlm.nih.gov/genbank) with the following accession numbers: MH920378-MH920435.

### Statistical analysis

Correlation analysis was performed using the Spearman rank test and linear regression. We calculated the intraclass correlation coefficient (ICC). To determine differences between two viral load assays, the Bland-Altman plot method^44^ was used. The clinically relevant difference between two VL measurements was considered at 0.5 log_10_ cp/ml, as described previously^45–47^. For all analysis, 95% confidence intervals were considered. All statistical analyses were performed using Excel, STATA v11 and GraphPad Prism 6.

### Ethical aspects

The project was approved by the Human Subjects Review Committees at Monkole Hospital/University of Kinshasa (Kinshasa, DRC), University Hospital Ramón y Cajal (Madrid, Spain) and University of Navarra (Pamplona, Spain). Informed consent of enrolled adults and of parents or guardians of enrolled children was obtained. All methods were carried out in accordance with relevant guidelines and regulations.

## Results

### High percentage of false positive diagnosis by rapid serological testing in DRC and delay in infant HIV diagnosis

We evaluated the HIV-1 quantification efficacy of different molecular assays (POC and non-POC) in dried blood carrying different HIV-1 non-B subtypes and complex recombinants, mainly URF. For that purpose, DBS were collected from 85 children (60 HIV-uninfected, 18 HIV-infected, and 7 HIV-exposed) and 75 HIV-infected adults from Kinshasa (DRC) during 2016. The main characteristics of the study subjects are recorded in Table 2. The 160 study subjects were mainly seropositive by rapid serological tests in DRC (58.75%), female (57.5%) and antiretroviral experienced (46.2%). The mean age for HIV diagnosis in DRC was 8.1 (SD 5.38) years old in children and 40.4 (SD 12.13) in adults. The mean age at DBS collection was 9.8 (SD 5.11) years old in children and 46.5 (SD 12.27) in adults. All but one children were born in Kinshasa, 8.2% were orphaned and 6 presented HIV/ *Mycobacterium tuberculosis* confection. Most adults were female (70.7%)

**Table 2:** Characteristics of study population from Kinshasa (DRC) with collected DBS in 2016.

	Children	Adults	Total (%)
Number	85 (100%)	75 (100%)	160 (100%)
Gender (male)	46 (54.1%)	22 (29.3%)	68 (42.5%)
**Mean age**			
At DBS collection (range)	9.8 (0–18)	46.5 (24.8–73)	26.5 (0–73)
At HIV diagnosis at DRC (range)	8.1 (0–16)	40.4 (0–65.3)	33.7 (0–65.3)
**HIV positive status**			
By rapid testing in DRC	18 (21.2%)	74 (98.7%)	92 (57.5%)
by molecular testing and serology in Spain	14 (16.5%)	70 (93.3%)	84 (52.5%)
False positive HIV diagnosis in DRC	4 (4.7%)	4 (5.3%)	8 (5%)
**ART exposure among 92 HIV**+ **diagnosed in DRC**			
ART naïve	2	10	13
ART	16*	58*	74
Unknown	0	7	5
**ART exposure among 84 with confirmed diagnosis in Spain**			
ART naïve	1	9	10
ART	13	56	69
Unknown	0	5	5
**HIV-1 viraemia among 69 confirmed HIV**+ **under ART in DRC**			
Not detected only by Roche VL	0	1	1
Not detected only by Xpert VL	0	4	4
Not detected by both Roche and Xpert VL	3	5	8
>1,000 cp/dot by Xpert VL	3	12	15
>1,000 cp/ml by Xpert VL*	12	52	64
>1,000 cp/dot by Roche VL	6	12	18
>1,000 cp/ml by Roche VL*	12	32	44
**Number of different ART regimens among 69 treated in DRC**			
1	10 (76.9%)	34 (60.7%)	44 (63.8%)
2	2 (15.4%)	14 (25%)	16 (23.2%)
3	0	7 (12.5%)	7 (10.1%)
4	1 (7.7%)	1 (1.8%)	2 (2.9%)
**NRTI experienced**			
3TC	13	58	71
AZT	10	46	56
TDF	5	27	32
DDI	0	3	3
ABC	0	4	4
**NNRTI experience**			
NVP	10	43	53
EFV	6	29	35
**PI experience**			
LPV/r	1	7	8
HIV+ subjects with available pol HIV-1 sequences	13 (92.8%)	45 (64.3%)	58/(69%)
**HIV-1 variant by phy**			
Non-B subtypes	2/13 (15.4%)	4/45 (8.9%)	6 (10.3%)
CRF	2/13 (15.4%)	8/45 (17.8%)	10 (17.3%)
URF	9/13 (69.2%)	33/45 (73.3%)	42 (72.4%)

DRC, Democratic Republic of Congo; DBS, dried blood Spot; ART, antiretroviral treatment; NTRI, nucleoside transcriptase reverse inhibitor; NNRTI, non-NRTI; PI, Protease inhibitor; 3TC, Lamivudine; AZT, Zidovudine; TDF, Tenofovir; DDI, Didanosine; ABC, Abacavir; NVP, Nevirapine; EFV, Efavirez; LVP/r, Lopinavir/Ritonavir; Phy, phylogenetic analysis; CRF, circulating recombinant form; URF, unique recombinant form. *Corrected cp/ml plasma considering hematocrit. Among 74 treated subjects in DRC, 3 were false positive children and 2 false positive adults.

Among the 92 children and adults diagnosed as HIV positive in the local laboratory in Congo by rapid tests and 80.4% were antiretroviral (ARV) experienced at sampling. The remaining were ART-naive or with unknown treatment data. NRTI and NNRTI were the most used ARVs in the study cohort, Zidovudine + Lamivudine + Nevirapine being prescribed in 60 treated patients (13 children and 47 adults), mainly as first line therapy (50 cases). Only 8 patients received protease inhibitors (PIs) based treatment with Lopinavir/Ritonavir, while integrase inhibitor use was absent among study subjects. (Table 2).

HIV-1 infection was confirmed in 16.5% of 85 children by Xpert Qual and in 93.3% of 75 HIV+ adults diagnosed in DRC by both Xpert VL and Roche VL. However, we identified false positive HIV diagnosis in DRC after following rapid serology testing algorithm in 4 adults (range 23.8–28.5 years age) and in 4 children (range 5.3–13.1 years age), and 5 of them (2 adults, 3 children) were under unnecessary ART for a mean time of 3.9 years (Table 3). Xpert Qual confirmed the absence of HIV-1 infection in 4 DBS from children erroneously diagnosed as HIV positive in the DRC, as in other 8 HIV negative cases providing undetermined HIV status by Geenius BioRad. The 4 false positive in adults provided negative results by two VL assays (Roche and Xpert), three of them provided negative result by two 4^th^ generation immunoassays (Roche Elecsys® *HIV combi PT and* bioMerieux *VIDAS*^®^ *HIV Duo Quick)*, and the remaining case only by VIDAS assay. Thus, the rate of false positive HIV diagnosis among pediatric HIV-exposed population was 22.2% (4/18) and 5.3% (4/75) for HIV-infected adults.

**Table 3:** Eight false HIV diagnosis in DRC using rapid serological testing.

	IDSpain	ARV experience at sampling	Time under ART (m)	Democratic Republic of the Congo					Spain							
				Determine	Uni-Gold	Double Check	Definitive diagnosis	Age at diagnosis (y)	ElecsysRoche	Vidas Duo Quick	BioRad Geenius	Xpert Qual	Roche VL	Xpert VL	Definitive diagnosis	Age at diagnosis (y)
*Children*	P6	AZT + 3TC + NVP	96	Pos	Pos	Pos	Pos	unknown	—	—	Neg	Neg	—	—	HIV-	9.11
	P12	AZT + 3TC + NVP	21	Pos	Pos	Pos	Pos	0.55	—	—	Neg	Neg	—	—	HIV-	5.36
	P14	AZT + 3TC + NVP	22.4	Pos	Pos	Pos	Pos	3.57	—	—	Ind	Neg	—	—	HIV-	8.64
	N18	Naive	0	Pos	Pos	Pos	Pos	6.77	—	—	Ind	Neg	—	—	HIV-	13.17
Adults	CUN84	AZT + 3TC + NVP	176.4	Pos	Pos	—	Pos	unknown	Pos	Neg	Neg	—	Neg	Neg	HIV-	24.7
	CUN33	Naive	0	Pos	Pos	—	Pos	0 (birth)	Neg	Neg	Neg	—	Neg	Neg	HIV-	28.7
	CUN41	AZT + 3TC + EFV	87	Pos	Pos	—	Pos	28.5	Neg	Neg	Neg	—	Neg	Neg	HIV-	unknown
	CUN109	Naive	0	Pos	Pos	—	Pos	51	Neg	Neg	Neg	—	Neg	Neg	HIV-	29.07

HIV testing in DRC: Determine, rapid test Determine™ HIV-1/2 Ag/Ab (Alere); Uni-Gold, Uni-Gold HIV (Trinity Biotech) and Double-Check, Double-Check Gold HIV 1&2 (Orgenics). HIV testing in Spain: Elecsys Roche, 4^th^ gen immunoassay Elecsys® HIV combi PT (Roche); *VIDAS DUO Quick*, 4^th^ gen immunoassay VIDAS® HIV Duo Quick (bioMerieux); BioRad Geenius^TM^ HIV-1/2; Xpert Qual, Cepheid Xpert Qual; Xpert VL, Cepheid Xpert HIV-1 VL; Roche VL, COBAS® AmpliPrep/COBAS® TaqMan® HIV-1 Test v2.0. *ARV*, *antiretroviral drugs; AZT*, *Zidovudine; 3TC*, *Lamivudine; NVP*, *Nevirapine; EFV*, *Efavirenz; m*, *months; y*, *years; VL*, *viral load; dash*, *not done; Ind*, *indeterminate; Neg*, *HIV negative; Pos*, *HIV positive*.

### Successful detection of HIV-1 variants in Kinshasa by Xpert VL and Roche VL

Among the 84 HIV+ total samples selected after excluding those 8 DBS samples determined to be false positives, 95.2% could be detected by Xpert VL and 97.6% by Roche VL, the remaining being undetected (Table 4). Among those specimens with detectable HIV RNA, Xpert VL *vs*. Roche VL could quantify and provide HIV-1 viraemia values (≥40 *vs*. ≥20 cp/dot or ≥936–1078 *vs*. ≥468–539 cp/ml plasma depending on hematocrit) for 65 (77.4%) *vs*. 66 (78.6%) DBS samples, respectively. Most (92.9%) of 14 HIV+ children presented quantifiable viraemia by both Xpert-VL (≥40 cp/dot) and Roche-VL (≥20 cp/dot) assays and 74.3% or 72.8% of 70 HIV+ adults by Xpert or Roche, respectively. Two specimens not detected by Roche were detected by Xpert (<40 cp/ml), while 4 specimens below limit of detection of Xpert could be only detected by Roche, with lower detection limit (<20 cp/ml) (Table 4).

**Table 4:** HIV-1 VL quantification in 84 HIV-1+ DBS (14 HIV+ children and 70 HIV+ adults) using two molecular assays.

			Xpert VL (POC assay)					
			Not detected	Detected not quantified <40 cp/dot	Quantified ≥40 cp/dot			No. DBS
					40–1,000 cp/dot	>1,000 cp/dot	Ct mean [range]	
Roche VL (Non-POC assay)	Not detected		—	2	0	0	0	2
	Detected not quantified <20 cp/dot		4	9	3	0	34.1 [30.1–36.6]	16
	Quantified (≥20 cp/dot)	≥20–39 cp/dot	0	3	7	0	32.5 [31.8–34.2]	10
		40–1,000 cp/dot	0	1	25	2	30.3 [28.2–32.8]	28
		>1,000 cp/dot	0	0	3	25	25.2 [20.3–27.4]	28
		Ct mean [range]	0	39.6 [33.2–42.6]	36.3 [33.9–41.8]	30.2 [23.6–33.6]	—	—
	No. DBS		4	15	38	27	—	84
Available *pol* HIV-1 sequences			nd	4 (26.7%)	27 (71%)	27 (100%)		58 (69%)
Carrying complex recombinants (CRF + URF)			nd	4	23	25	—	52
Carrying non-B variants			nd	0	4	2	—	6

No., number; VL, HIV-1 viral load POC, point of care; DBS, dried blood sample; cp/dot, HIV-1 RNA copies per DBS dot; nd, not available sequences due to low VL. Ct, VL assay-specific cycle threshold, which inversely correlates with the starting concentration of the viral genome in infected specimen.

### High correlation among Xpert and Roche VL assays using DBS

**Figure 1:**
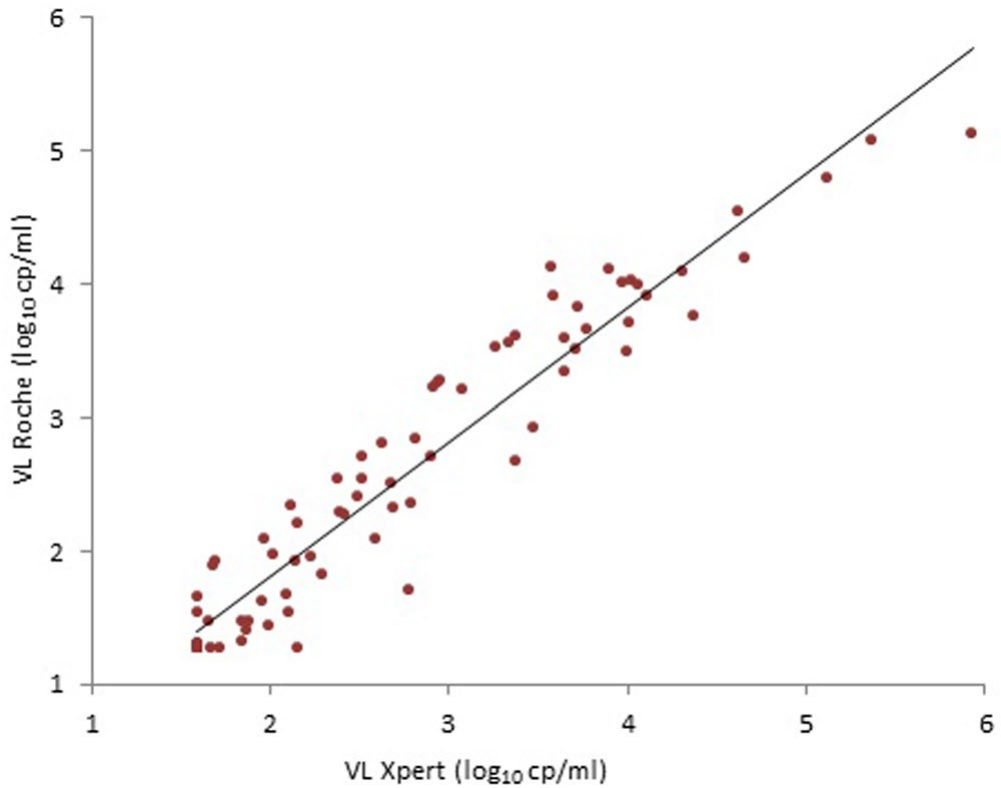
Correlation between Xpert VL and Roche VL assays in 78 HIV+ samples with quantified VL. Scatter plot with a simple linear regression analysis of 78 samples (14 children + 64 adults) which were quantified (VL of **≥**40 or ≥20 cp/ml) by both assays. Graphic using log of direct VL from in one DBS dot (HIV-1 RNA copies per dot). VL, Viral load.

Viral Load results within the quantification range of both assays were available for 78 (92.8%) of 84 HIV+ patients. The POC Cepheid Xpert HIV-1 VL assay showed excellent agreement (ICC = 1) with Roche VL for HIV-RNA quantification. A high and significant correlation was observed among both VL assays (R^2^ = 0.89, *P* < 0.001), as shows the estimated regression line (Fig. 1). However, Ct values for HIV-1 quantification were VL assay dependent when quantifying the 78 DBS detected by both VL assays.

Xpert VL provided higher mean Ct (34.75 ± 7.7, range 23.6–42.6) than Roche VL (29.4 ± 3.77, range 20.3–36.6) in the panel, resulting in a mean Xpert VL of 4.22 log_10_ cp/ml ± SD 1.06 (2.76 log_10_ cp/dot ± SD 1.16) and mean Roche VL of 4.04 log_10_ cp/ml ± SD 1.12 (2.57 log_10_ cp/dot ± SD 1.12).

**Figure 2:**
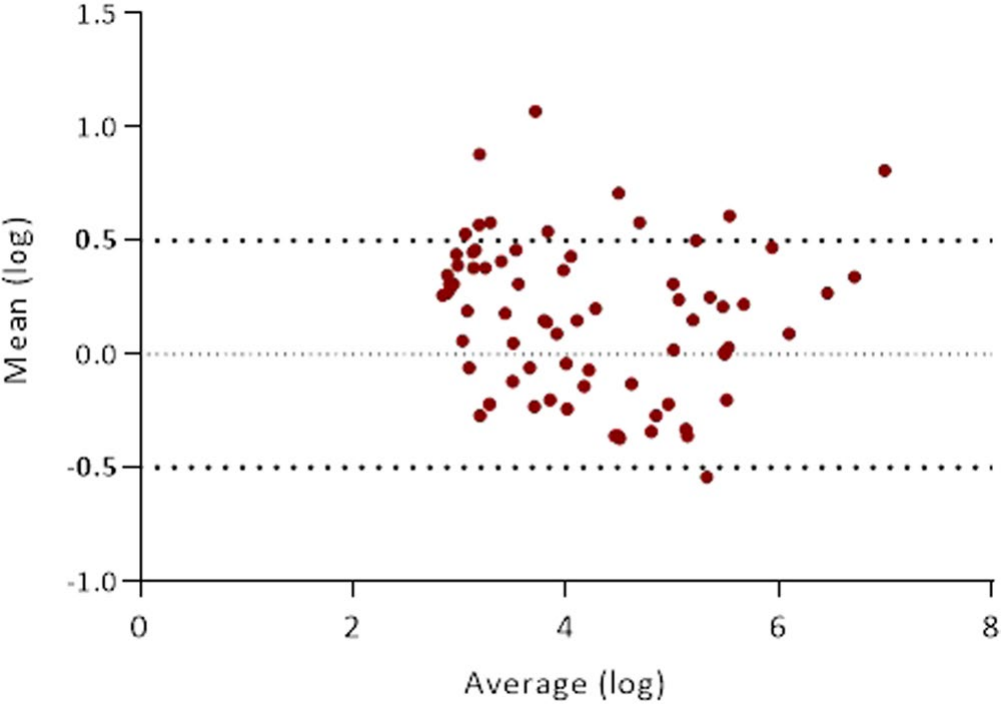
Bland-Altman analysis showing difference *vs*. average viral load comparing Xpert VL and. Roche VL in 78 HIV+ quantified by both assays. HIV+ samples from 78 patients (14 children, 64 adults) quantified by both assays.

**Figure 3:**
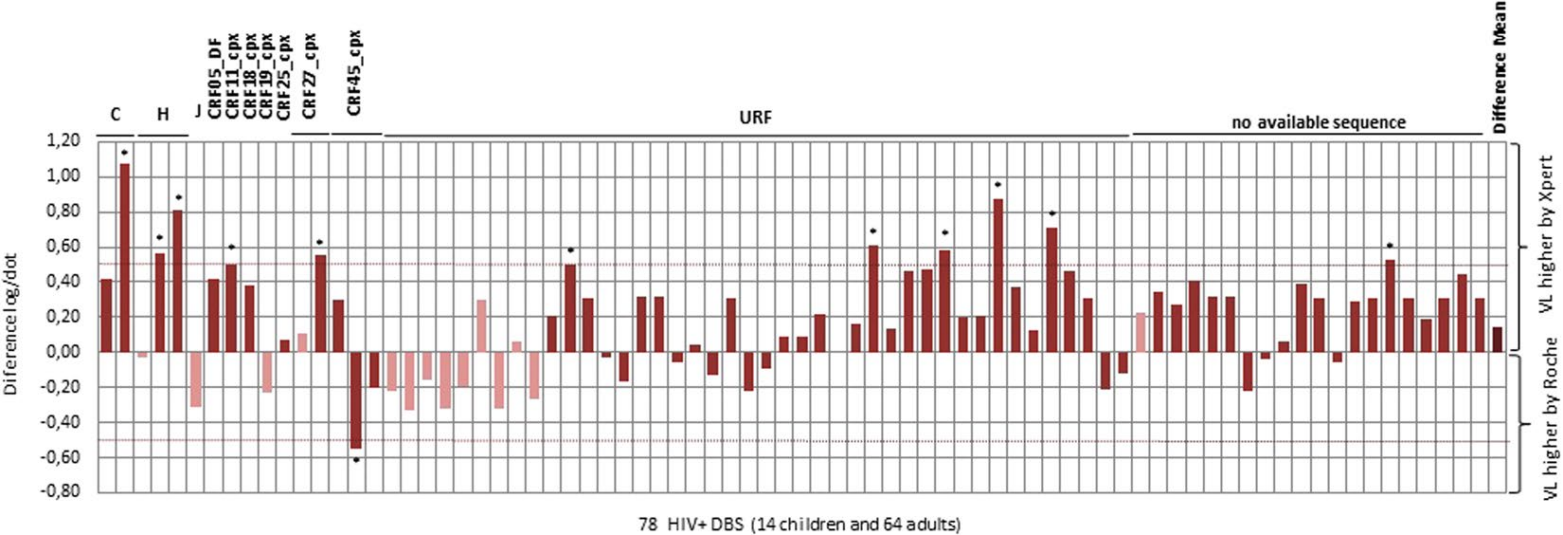
HIV-1 viraemia differences by Xpert VL *vs*. Roche VL in 78 HIV+ DBS quantified by both assays and HIV-1 variants in 58 samples with available sequence. Light color in pediatric samples. VL, viral load; 58 non-B variants infecting study population: 2C, 3H, 1J, 1CRF05_DF, 1CRF11_cpx, 1 CRF18_cpx, 1CRF19_cpx, 1 CRF25_cpx, 2 CRF27_cpx, 3 CRF45_cpx. The absence of bar in one URF indicates the same VL values using both assays. CRF, circulating recombinant form; URF, unique recombinant form. *Viral load differences >0.5 log.

The similarities between both VL assays were evaluated by the Bland-Altman plot method (Fig. 2). HIV-1 VL overestimation by one of the two assays (Xpert VL or Roche VL) was observed in all but one specimen among the 78 DBS, although the difference was below a clinically relevant threshold of 0.5 log_10_ cp/ml in most cases (84.6%). The POC Xpert HIV-1 VL assay tended to overestimate HIV-1 VL in 69.2% samples, and the non-POC Roche VL in 29.5% specimens (Fig. 3, Table 4). The overall mean difference in the HIV-1 RNA values obtained by Xpert VL assay and Roche VL was 0.30 log_10_ cp/dot (95% CI: 0.26 to 0.35 log_10_ cp/dot) (*P* < 0.001). However, clinical relevant differences (≥0.5 log VL) ranging from −0.55 to 1.07 were observed in 12 (15.4%) of 78 DBS specimens with VL above detection limit by both assays (Figs 2 and 3), differing across samples and assays. Eleven cases corresponded to Xpert VL use, while only one to Roche VL testing (Table 4).

### High percentage of complex recombinants in DRC and impact in VL quantification

HIV-1 *pol* sequence was recovered from 58 of 84 HIV-1 + individuals (13 children and 45 adults) and studied by phylogeny. Among all 58 viral sequences obtained, we identified 6 (10.4%) non-B subtypes (2C, 3H, 1J), ten (17.2%) CRF (1 CRF05_DF, 1 CRF11_cpx, 1 CRF18_cpx, 1 CRF19_cpx, 1 CRF25_cpx, 2 CRF27_cpx, 3 CRF45_cpx) and 42 (72.4%) URF. Thus, most (89.6%) of 58 obtained HIV-1 sequences were URF or CRF recombinants (Table 2). Neither HIV-2 nor non-M group infections were found in our study population. Both VL tests were able to detect and quantify all variants, including CRF and URF recombinants of the study cohort (Table 4).

The impact of each complex recombinant on VL quantification was unclear (Table 4, Supplementary Table 1, and Fig. 3). Among 42 URF detected, 61.9% provided higher VL by Xpert VL and 35.7% by Roche VL. Among 10 samples ascribed to 7 different CRF, 70% showed higher viraemia by Xpert VL and 30% by Roche VL. Subtypes C and H displayed higher viraemia values by Xpert VL and subtype J by Roche VL.

## Discussion

POC test use can improve the clinical management of HIV-infected infants and adults and reduce the delay in diagnosis and in ART failure identification^14^. Early infant diagnosis is a WHO priority^14,48^, since it allows early ART to be established and reduces irreversible damage to central nervous and immune systems, viral reservoirs, as well as HIV transmission and morbidity/mortality associated with HIV-1 infection^49^. A correct early HIV diagnosis is also essential, since false positive HIV tests might result in unnecessary antiretroviral treatment and psychological distress in falsely diagnosed individuals and families^50^. The use of DBS has been proposed as an alternative sample to plasma/serum, easier to be collected, stored and shipped, very convenient in limited resource countries^15,33,37,51^. The aim of this study was to evaluate the performance of two POC HIV-1 assays (Xpert-VL and Xpert Qual) for HIV detection and/or quantification using DBS in the DRC, a country with a high HIV-1 diversity including a high prevalence of complex recombinants, mainly URFs.

Although POC molecular testing prevents inappropriate HIV serological diagnosis and is cost-effective^52^, it is not yet globally adopted in all EID or adult programs^48^. In the DRC, the *National Program of fight against HIV-AIDS (PNLS*) recommends performing EID 0–2 days after birth, but POC assays for EID have not been implemented yet within the clinical routine of HIV-exposed infants.

We have evaluated the clinical impact of the lack of routine EID molecular testing in HIV-exposed newborns shortly after birth and of confirmatory serological testing in older children and adults in a cohort in Kinshasa. We have found false HIV diagnoses among 5% of the study participants that lead to unnecessary ART in five HIV uninfected subjects. The high prevalence of false positive diagnosis among HIV-exposed infants less than 18 years old could be explained by the long delay in PCR results from a centralized national laboratory, which were only available at the clinical center in Kinshasa 6 months after the original HIV serological test. Wrong diagnoses may also be a consequence of the absence of a confirmatory molecular test with a second new sample as recommended by WHO^14^ for infants. In older children and adults, false HIV diagnosis can be due to the local absence of confirmatory serological analysis and the exclusive use of serological rapid testing for HIV diagnosis. Although rapid immunochromatographic test for HIV are recommended in low income countries^53^, the low HIV prevalence among the general population in the DRC (0.7%)^4^ may be associated with a lower positive predictive value for these methods. In addition, rapid HIV testing is not appropriate for acute infection diagnosis^53^. Another aspect that could influence a misdiagnosis is the fact that a subjective reading of rapid HIV tests may speed up the communication of false positive results. The high mean age at HIV diagnosis in DRC in children (8.1 years) from the study cohort would suggest HIV diagnosis delay, since most of them acquired HIV infection by vertical route according to clinical reports.

Results also show that Xpert and Roche molecular tests were superior to fourth generation serological screening assays to identify HIV infection in HIV-exposed children older than 18 months and adults. Moreover, Xpert resolved HIV status and rule out HIV infection in DBS from 10 individuals with undetermined results by Geenius confirmatory assay, in agreement with recent studies^54^. It could be due to the fact that Xpert HIV-1 Qual can detect HIV-1 infections up to 7–10 days before seroconversion, an average of nine days earlier than a panel of HIV-1 antibody tests and five days earlier than a panel of HIV-1 antigen p24 tests^34^. Xpert® HIV-1 Qual may play a role in the diagnosis of HIV, either in EID, as a confirmatory test after antibody-based testing, or for the detection of acute HIV infection in antibody negative patients recently infected^55^.

POC Xpert VL and non-POC VL Roche assays were also suitable in quantifying VL using DBS in the DRC. A high percentage of subjects under ART in the DRC showed VL levels above 1,000 cp/ml by Xpert (92.7%) or Roche (63.8%) (Table 1), the VL threshold defined by WHO to confirm virological failure in low- and middle-income countries and when using DBS in adults and children^37^. This is the optimal threshold for presenting the lowest percentage of misclassification compared with higher thresholds^51^. In fact, most patients in the study had a clinical suspicion of virological failure. However, the prevalence of ART failure was overestimated using Roche VL and this threshold, resulting less specific than other VL assays using DBS, as described in previous studies^51,56^.

Despite the good correlation in VL quantification using Xpert VL with the no-POC Roche VL assay (R^2^ = 0.89), still 15.4% of 78 samples with VL within the quantification range of both assays presented clinically significant VL differences above 0.5 log_10_ cp/ml, according to previous studies^16,23,57^. Thus, we recommend the expanded use of VL in the DRC for an early detection of virological failure as well as the use of the same VL technique for each patient during ART monitoring to reduce potential assay-associated viraemia overestimations, which could be interpreted as virological failure events. This could reduce unnecessary ART regimen switches in these patients, favoring an early clinical response by reinforcing adherence or changing ART regimen if resistant variants are detected before clinical symptoms associated with treatment failure appear. The achievement of the 90-90-90 UNAIDS objectives depend on HIV monitoring, otherwise a future epidemic of HIV resistant strains may occur and delay these objectives in Sub-Saharan Africa^58^. The finding of assay dependent Ct values for HIV-1 quantification reinforces the risk of establishing a standard Ct cutoff as accurate threshold value to differentiate virological failures in subjects under ART.

The continuous evolution of HIV can hinder diagnosis and complicate clinical practice^59^. Thus, one of the main challenges for molecular diagnostic and VL assays is to detect and/or quantify different HIV-1 variants correctly. According to the manufacturer’s information, the Xpert® HIV-1 Qual assay has been validated for specimens including groups N, O and M (9 subtypes and recombinants A/E, A/B and AG/GH)^60^ and the Xpert VL for groups N, O and M (9 subtypes, CRF01_AE, CRF02_AG, and CRF03_AB)^61^. However, most 98 HIV-1 CRF^43^ and complex unique recombinant forms (URF) have not been validated yet. Roche VL was evaluated by analysis of HIV-1 group O and group M subtypes A through H from cell culture origin^36,62^, although it was also able to quantify a number of CRFs^16^. However, none of them has been evaluated across a large panel of URF variants, as we reported. We demonstrate that POC-Xpert assays and VL and Roche-VL can successfully detect and quantify complex recombinants in *pol*.

We also provide new data related to the HIV molecular epidemiology in Kinshasa, reporting an extremely high rate of unique inter-subtype recombinants in recently infected populations, although URF prevalence could be underestimated since HIV-1 variants were characterized considering *pol* gene but not the complete genome. Other authors have also highlighted the extreme diversity of HIV strains circulating in the country with a high presence of URFs and different CRFs, together with a low presence of subtype C^28,31^.

Some studies have reported clinically-significant differences (>0.5 log) in VL quantification across techniques with the same non-B strains^23,63–65^. These results would suggest different rates of detection across assays due to genetic variability in the HIV-1 gene region targeted by the assays in incorrectly quantified samples, as previously demonstrated for a CRF02_AG variant^63^. Previous evaluations supported the efficacy of Xpert technology for the detection and quantification of HIV-1 non-B variants^64,66–68^, highlighting Xpert utility in areas with a high genetic variability of HIV, such as Sub-Saharan Africa^57,69–72^.

POC Xpert technology for diagnosis of other diseases such as tuberculosis^73^, shigellosis^74^ or Monkeypox virus^75^ has already been used in the DRC. The National HIV/AIDS Program in the DRC plans to use the existing Xpert machines for HIV EID testing and VL monitoring in order to improve the clinical care of HIV-infected children and adults. Our results demonstrate that Xpert performance is adequate for testing HIV-1 variants currently circulating in the DRC. Thus, due to simplicity, rapid results and good performance, POC Xpert HIV-1 can be useful in the decentralization of EID and VL monitoring from specialized laboratories in the DRC to local hospitals and clinics within their routine clinical care. This will help to reach the ambitious 90-90-90 goals in the country. We also confirmed that DBS could be a suitable sample for Xpert use in the DRC, requiring a minimum volume of blood, favoring molecular testing in infants and low-weight children. In addition, DBS samples offer additional advantages, they are not considered biohazardous once dried and are not as time and temperature sensitive as plasma specimens^37^.

An important limitation of this study is that we did not compare DBS to plasma due to the lack of paired plasma/DBS specimens collected for each subject in the study population. Moreover, due to the design of the study and sample size, we could not determine the statistical power of possible performance differences across assays in each specific HIV-1 variant. Finally, we could not explore the effect of DBS lysis buffer in viraemia quantification, which could influence DBS VL results according to previous reports^76^. The main strength of our study is that it shows the first results confirming the utility of POC Xpert HIV-1 tests and Roche VL platform for early HIV-1 diagnosis and for VL quantification of complex recombinants (mainly URF) currently circulating in Kinshasa, the epicenter of HIV-1 group M epidemic and where a large number of complex recombinants cocirculate. To our knowledge, there are no previous studies that have included a large panel of different HIV-1 complex recombinants characterized by phylogenetic analysis during Xpert-POC HIV-1 evaluation for EID and VL. We also report some of the current limitations of HIV diagnosis and monitoring in DRC. Since Xpert assays and DBS use can improve early diagnosis in HIV-exposed infants and early detection of ART failures in countries with complex HIV-1 recombinants and limited infrastructures, as in the DRC, our results could have a direct clinical impact in global HIV diagnosis and monitoring to reach early the 90-90-90 objectives.

## Supplementary information

Supplementary Table 1
