# Supplementary material for: Utility Of POC Xpert HIV-1 Tests For Detection-Quantification Of Complex HIV Recombinants Using Dried Blood Spots From Kinshasa, D. R. Congo
Source: Sci Rep. 2019 Apr 5;9:5679. doi: 10.1038/s41598-019-41963-y (PMC6450884; doi:10.1038/s41598-019-41963-y)
Supplement: Supplementary file 1 — Supplementary Table 1 [file 41598_2019_41963_MOESM1_ESM.pdf]

UTILITY OF POC XPERT HIV-1 TESTS FOR DETECTION-QUANTIFICATION OF COMPLEX HIV RECOMBINANTS USING DRIED BLOOD SPOTS FROM KINSHASA, D.R.CONGO

Marina Rubio-Garrido[1], Adolphe Ndarabu[2], Gabriel Reina*[3], David Barquín[3], Mirian Fernández-Alonso[3], Silvia Carlos[4] and África Holguín[1].

[1] HIV-1 Molecular Epidemiology Laboratory, Microbiology and Parasitology Department, Hospital Ramón y Cajal-IRYCIS and CIBER-ESP, Madrid (28034), Spain.

[2] Monkole Hospital, Kinshasa, Democratic Republic of Congo.

[3] Microbiology Department, Clínica Universidad de Navarra. Navarra Institute for Health Research (IdiSNA). Institute of Tropical Health, Universidad de Navarra (ISTUN), Pamplona (31008), Spain.

[4] Department of Preventive Medicine and Public Health, Universidad de Navarra, Pamplona (31008), Spain. Navarra Institute for Health Research (IdiSNA). Institute of Culture and Society (ICS), Institute of Tropical Health, Universidad de Navarra (ISTUN), Pamplona (31008), Spain.

* Correspondence: *gabi@unav.es*

Supplementary Table 1. Viraemia overestimation considering VL assays and HIV-1 variants defined by phy. VL, viral load; phy, phylogenetic analysis; DBS, dried blood spot; CRF, circulating recombinant form; URF, unique recombinant form.

VL in DBS	Number of 78 DBS	HIV-1 variants
Higher by Xpert VL	54 (69.2%)	2C, 2H, 1CRF05_DF, 1CRF11_cpx, 1CRF18_cpx, 1CRF25_cpx, 2CRF27_cpx, 1CR45_cpx, 26URF, 17 without sequence
Higher by Xpert VL (>0.5 log)	11 total (14.1%) 11/54 overestimated (20.3%)	1C, 2H, 1CRF11_cpx, 1CRF27_cpx, 5URF, 1 without sequence
Higher by Roche VL	23 (29.5%)	1H , 1J, 1CRF19_cpx, 2CR45_cpx, 15 URFs, 3 without sequence
Higher by Roche VL (>0.5 log)	1 total (1.3%) 1/23 overestimated (4.3%)	1CRF45_cpx
Total cases with >0.5 log VL differences across assays	12 (15.4%)	1C, 2H, 1CRF11_cpx, 1CRF27_cpx, 1CRF45_cpx, 5URF, 1 without sequence
